# Motor function is the primary driver of the associations of sarcopenia and physical frailty with adverse health outcomes in community-dwelling older adults

**DOI:** 10.1371/journal.pone.0245680

**Published:** 2021-02-02

**Authors:** Aron S. Buchman, Sue E. Leurgans, Tianhao Wang, Michal Schnaider-Beeri, Puja Agarwal, Robert J. Dawe, Osvaldo Delbono, David A. Bennett

**Affiliations:** 1 Rush Alzheimer’s Disease Center, Rush University Medical Center, Chicago, IL, United States of America; 2 Department of Neurological Sciences, Rush University Medical Center, Chicago, IL, United States of America; 3 Icahn School of Medicine at Mount Sinai, New York, NY, United States of America; 4 Joseph Sagol School of Neuroscience, Tel Aviv University, Tel Aviv, Israel; 5 Department of Internal Medicine, Rush University Medical Center, Chicago, IL, United States of America; 6 Department of Diagnostic Radiology and Nuclear Medicine, Rush University Medical Center, Chicago, IL, United States of America; 7 Section of Gerontology, Wake Forest School of Medicine, Winston-Salem, NC, United States of America; Universita degli Studi di Napoli Federico II, ITALY

## Abstract

**Background:**

This study tested the hypothesis that sarcopenia and its constituent components, reduced lean muscle mass and impaired motor function, are associated with reduced survival and increased risk of incident disabilities.

**Methods:**

1466 community-dwelling older adults underwent assessment of muscle mass with bioelectrical impedance analysis (BIA), grip strength, gait speed and other components of physical frailty and annual self-report assessments of disability. We used Cox proportional hazards models that controlled for age, sex, race, education and height to examine the associations of a continuous sarcopenia metric with the hazard of death and incident disabilities.

**Results:**

Mean baseline age was about 80 years old and follow-up was 5.5 years. In a proportional hazards model controlling for age, sex, race, education and baseline sarcopenia, each 1-SD higher score on a continuous sarcopenia scale was associated with lower hazards of death (HR 0.70, 95%CI [0.62, 0.78]), incident IADL (HR 0.80,95%CI [0.70, 0.93]), incident ADL disability (HR 0.80 95%CI [71, 91]) and incident mobility disability (HR 0.81, 95%CI [0.70, 0.93]). Further analyses suggest that grip strength and gait speed rather than muscle mass drive the associations with all four adverse health outcomes. Similar findings were observed when controlling for additional measures used to assess physical frailty including BMI, fatigue and physical activity.

**Conclusions:**

Motor function is the primary driver of the associations of sarcopenia and physical frailty with diverse adverse health outcomes. Further work is needed to identify other facets of muscle structure and motor function which together can identify adults at risk for specific adverse health outcomes.

## Introduction

Aging is associated with degenerative and structural changes in muscle and body composition as well as other musculoskeletal elements. These degenerative changes may contribute to loss of muscle structure as well as degrade its function as the final effector of all motor behavior [1]. Concurrent changes in muscle structure and function have long been recognized as common elements of aging. Much research over the past two decades has suggested that combining indices of muscle structure using measures of lean muscle mass and muscle function assessed by grip strength into a single phenotype called sarcopenia may facilitate the identification of older adults at risk for adverse health outcomes [2].

In the past, measurements of lean muscle mass required instruments which were not portable making it difficult to assess sarcopenia across the full health range of older adults in community-based studies. Consensus developed recommending measurements of both lean muscle mass together with loss of muscle function assessed by grip strength and/or gait speed to identify sarcopenia [2]. During this period, portable devices based on measures of electrical bioimpendance became available and led to a wealth of information about sarcopenia in aging adults. These new data have led to a reassessment of the independent role of lean muscle mass metrics as part of the construct of sarcopenia as well as the recognition of the difficulties of using categorical measures for the capturing the heterogeneity of aging phenotypes in old age [3, 4].

Binary assessments of sarcopenia developed to date are utilized to estimate its prevalence, for the design of clinical trials and because of the ease of binary measures for decision making in the clinical setting. Prior work has shown that continuous composite measures may have improved metric properties for capturing the heterogeneity of aging phenotypes and have an important role for longitudinal modeling of the trajectories of aging motor phenotypes [5–7].

The current study, used clinical data from community-dwelling older adults participating in the Rush Memory and Aging Project to test the hypothesis that a composite measure of sarcopenia and its components, lean muscle mass and grip strength are independently associated with the hazard of mortality and incident disabilities [8]. Both sarcopenia and another common aging phenotype, physical frailty, are based on assessments of different aspects of muscle bulk and motor function [2, 9]. In further analyses, we examined the inter-relationship between the components used to construct these phenotypes, to test the hypothesis that lean muscle mass and other non-motor measures are independently associated with adverse health outcomes when controlling for motor function.

## Methods

### Participants

All participants were from the Rush Memory and Aging Project, a longitudinal clinical-pathologic investigation of chronic conditions of old age. Participants were recruited from more than 40 residential facilities across the metropolitan Chicago area, including subsidized senior housing facilities, retirement communities, and retirement homes, in addition to social service agencies and church groups. Participants agreed to annual detailed clinical evaluations (described below). All evaluations were performed at the parent facility or the participants’ homes to reduce burden and enhance follow-up participation. In addition, written informed consent and an Anatomical Gift Act for organ donation at the time of death was obtained from each study participant. The study was in accordance with the latest version of the Declaration of Helsinki and was approved by the Institutional Review Board of Rush University Medical Center. All data used in this study can be requested at www.radc.rush.edu.

Each person underwent a uniform structured clinical evaluation, which included a medical history, as well as assessment of physical and cognitive functions (see below). Follow-up clinical evaluations, identical in all essential details to the baseline examination, were performed at one-year intervals by examiners blinded to previously collected data. Baseline for these analyses was the first cycle with bioimpendance testing. Although MAP began in 1997, bioimpendance testing was not added until 2005. Thus, of 2116 participants who had completed MAP baseline when analysis was conducted, 1500 had valid bioimpendance and grip strength testing. Of those, 5 were missing longitudinal outcomes and 34 were missing one or more demographic covariates and were excluded. This left 1466 for these analyses, with a mean follow-up of 5.6 years (SD, 3.8 years).

### Adverse health outcomes

#### Mortality

When an autopsy is obtained, date of death is known. When no autopsy is obtained, we obtain information on date of death from a knowledgeable informant or from searches of public databases [10].

#### Disability

Was assessed annually via three self-report instruments. Instrumental activities of daily living (IADLs) were assessed using items adapted from the Duke Older Americans Resources and Services project, which assess eight activities: telephone use, meal preparation, money management, medication management, light and heavy housekeeping, shopping, and local travel [11]. Basic activities of daily living (ADLs) were assessed using a modified version of the ADL scale which assesses six activities: feeding, bathing, dressing, toileting, transferring, and walking across a small room [12]. Mobility disability was assessed using the Rosow-Breslau scale, which assesses three activities: walking up and down a flight of stairs, walking a half mile, and doing heavy housework like washing windows, walls, or floors [13].

For all three scales, participants were given the following response choices with regard to their ability to perform each of the above activities: no help, help, unable to do. For these analyses, a participant was considered disabled for the domain evaluated by the scale unless the participant reports that they can do all the activities without help.

### Assessment of sarcopenia

Our measure of sarcopenia was based on measures of lean muscle mass and motor function, and is compared to a common binary sarcopenia measure reported in consensus publications [14].

#### Muscle function

Grip strength (kg) was measured with the Jamar hydraulic hand dynamometer (Lafayette Instruments, Lafayette, IN). Two trials of isometric grip strength were obtained bilaterally and averaged to yield grip strength. Gait speed (m/s) was based on the self-paced time to walk eight feet [8].

#### Skeletal Muscle Mass (SMM)

Was based on bioelectrical impedance analysis (BIA) ohms recorded with Body Comp Scale BCS-1 series, which sends a weak alternating current through the body in order to probe the tissue’s overall resistance, or impedance, to that flow of electrons. This impedance is related to the total body water, which depends on the relative amounts of fat and muscle (Firmware version: 1.34) Company: Valhalla Scientific, Inc [15, 16]. Janssen et al derived a formula to convert BIA to estimated muscle mass from MRI evaluation; the expression is 0.401 times the product of the square of the height in centimeters and the reciprocal of the bioimpedance in ohms, with adjustments for age, sex, and race [15, 17].

#### Skeletal Muscle Mass Index (SMI)

Following the familiar conversion from total body mass to BMI, an SMI (skeletal muscle mass index) was computed by dividing the estimate of skeletal muscle mass by weight^2^. We computed this index for the Janssen formula for SMM as an intermediate step in deriving continuous and binary variables.

#### Continuous sarcopenia and binary sarcopenia

We derived a previously published binary variable utilizing sex-specific binary classifications of low or not low muscle mass (male <8.87; female <6.42) and grip strength (male <30kg; female <20kg) [14]. Many composite measures average several conceptually related measures to improve the metric properties of the resulting summary measure. Such approaches do not capture or represent the interactions among the individual component measures. The binary variable for the presence of sarcopenia requires both strength and muscle mass to be reduced. In contrast, individuals with either one or both measures above their respective cut-offs are considered non-sarcopenic. Thus, simply averaging strength and muscle mass together may not capture an interaction between strength and muscle mass.

**Fig 1:**
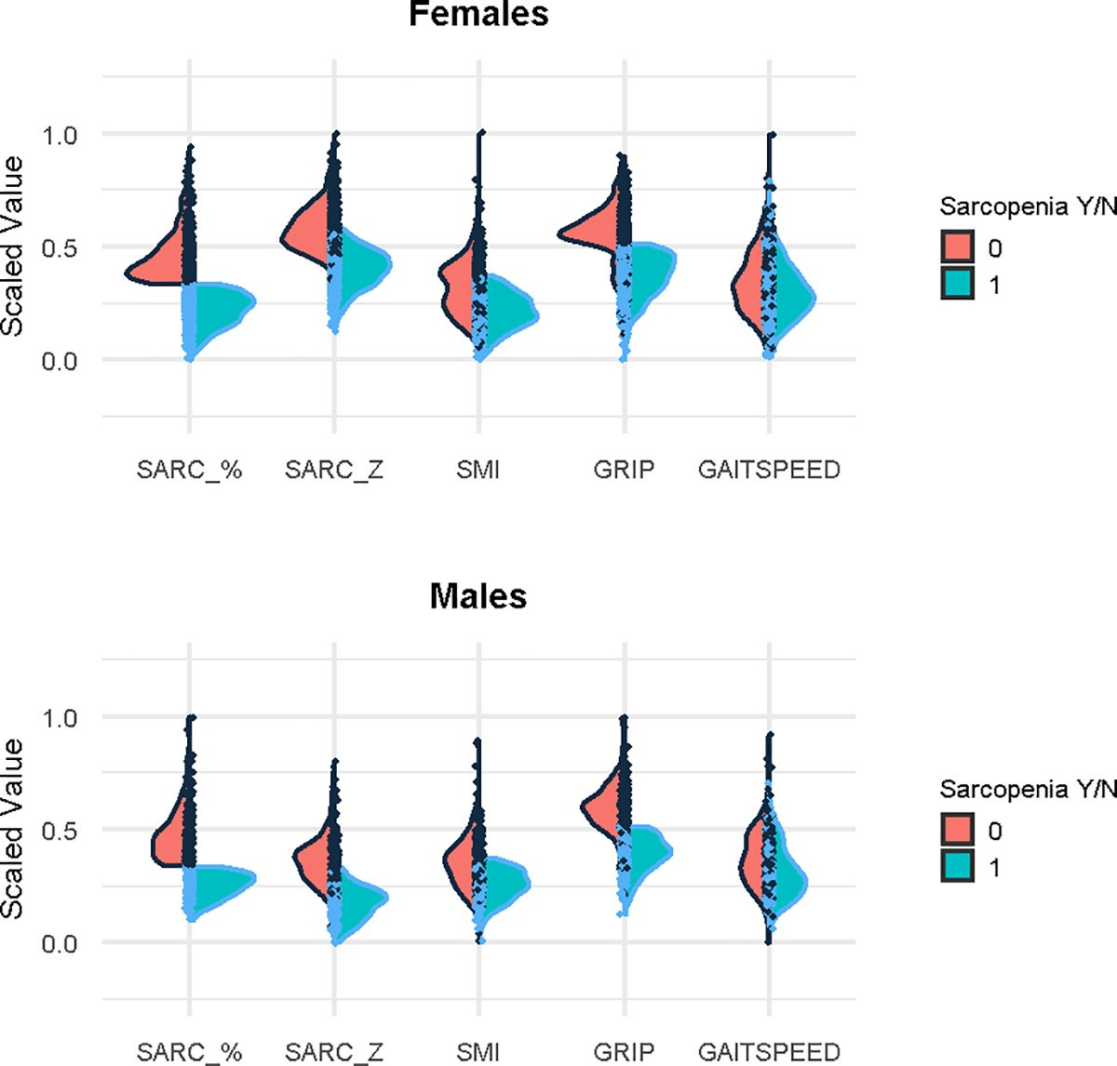
Heterogeneity of muscle mass and motor function in binary and composite sarcopenia metrics. Published binary sarcopenia (Sarcopenia Y/N) is based on sex-specific thresholds, so males and females are presented in separate panels. Each panel shows five variables with density estimates for the persons meeting (bluegreen) and not meeting (orange) criteria for binary sarcopenia juxtaposed to form figures that are referred to as violin plots. Each variable was scaled to range from 0 to 1; low scaled values in this figure represent greater sarcopenia. Continuous sarcopenia based on percentage (**Continuous Sarcopenia, Sarc%)** is on the far left and the composite sarcopenia average score (**Composite Sarcopenia, Sarc_z**) immediately to its right. These composite measures are followed by the two variables used to construct composite sarcopenia (skeletal muscle mass index (SMI) and grip strength). Gait speed, which is an alternative measure of motor function sometimes employed in constructions sarcopenia, is shown on the right. Review of the two panels in this figure confirms that sarcopenic persons had both weak grip and low SMI, with sharp limits for the bluegreen densities to the right of the vertical axes. It is evident that many persons with low SMI are not sarcopenic, because the red density (to the left of the vertical axes) overlaps the bluegreen density: these persons *a fortiori* do not have weak grip. Fig 1 also shows that the distribution of gait speed is similar for those with and without sarcopenia.

We extended the binary sarcopenia variable to a continuous sarcopenia variable by taking the maximum of skeletal muscle index and grip strength, each expressed as a percentage of the corresponding sex-specific threshold (**Fig 1**). Participants with a continuous sarcopenia measure < 100% are considered to manifest sarcopenia, as both SMI and grip strength are below their sex-specific cut-offs (**S1 Fig**). Lower values of continuous sarcopenia correspond to more severe sarcopenia. Participants whose continuous sarcopenia measure is at or above 100% must have at least one criterion at or above the threshold, so these participants are deemed non-sarcopenic [14]. See **S1 Method** for specific examples.

An alternative variable, a composite sarcopenia average variable, also described in the **S1 Method**, employed a standard approach for developing a composite metrics. Separately for men and for women, we centered and scaled both the muscle index and grip strength by (1) subtracting the corresponding mean at first muscle mass measurement, (2) dividing by the respective standard deviation to obtain z-scores, and (3) averaging the pair of z-scores for each person.

The continuous sarcopenia measure and the composite sarcopenia average measure are strongly correlated (r = 0.638, p<0.001). Our results using this alternative approach (**S1 Table**) were similar to those described in the main text using the continuous sarcopenia based on muscle mass and grip strength percentages.

### Other covariates and components of physical frailty

Sex, race and years of education were recorded at the baseline interview. Age in years was computed from self-reported date of birth and date of the clinical examination at which the strength measure was collected.

#### Physical frailty

In these analyses we also examined continuous measures of the five components which have been used to construct previously validated measures of physical frailty employed in this cohort and by other investigators [5, 9, 18–22]. In addition to grip strength, gait speed was based on the timed to walk 8 feet. Body mass index (BMI) was based on measured weight /height^2^. Two questions derived from a modified version of the Center for Epidemiologic Studies-Depression (CES-D) Scale were used to assess fatigue (questions 7 and 20): a) I felt that everything I did was an effort, and (b) I could not get “going” [9, 23]. Participants answered "yes" or "no" to each question, and the number of the two questions to which the participant answered “yes” provides the three-level variable we utilized. To compute a combined summary of these four measures, we computed z-scores based on MAP baseline mean and SD and averaged the four z-scores after multiplying suitable z-scores = by -1 so that higher scores of the composite frailty variable correspond to more severe physical frailty. To construct an ordinal measure of physical frailty employed by other investigators we dichotomized the four measures mentioned above as well as self-reported physical activity during the past two weeks based on 5 different types of activities as described in prior publications [24].

### Dietary assessment

Annual dietary assessments were begun in 2004 using a comprehensive 144 item modified Harvard semi-quantitative food frequency questionnaire (FFQ) that was validated for use in older Chicago community residents [25, 26]. The FFQ ascertained the usual intake frequency over the previous 12 months of various food items and dietary supplements. In the current analyses we used computed three measures including total daily calories, daily protein consumption and diet quality score.

To compute total daily calories (kcal) and protein (grams) intake, frequency consumption of each food was multiplied by calories/protein of natural portion size (e.g., one banana/ 1 bread) or the sex-specific mean portion sizes reported by the oldest men and women of national surveys and then summing over all items. Percentage calories from protein were also calculated (protein(grams)*4/total calories (kcal))*100).

Diet quality score is related to nutritional status and is a marker of metabolic health [27, 28]. To ascertain the quality of our participants' diet, we calculated the MIND (Mediterranean-DASH Intervention for Neurodegenerative Delay) diet score. In prior work in this cohort we have shown that a higher score is associated with a lower disability score [29]. The MIND diet is based on fifteen dietary components, including ten brain-healthy food groups (green leafy vegetables, other vegetables, nuts, berries, beans, whole grains, fish, poultry, olive oil, and wine) and five unhealthy food groups (red meats, butter, and stick margarine, cheese, pastries and sweets, and fried/fast food). Individual food groups were scored 0 (low adherence), 0.5, or 1 (high adherence) and then summed over all 15 dietary components for a score ranging from 0 to 15 as described previously [30].

### Analyses

We examined bivariate associations of sarcopenia with Pearson correlations. We employed a set of discrete-time Cox proportional hazards models to examine the association of continuous sarcopenia with incident adverse health outcomes. All models controlled for age, sex, education and race. Cox models used continuous time when the outcome was mortality.

Since continuous sarcopenia was constructed from muscle mass and grip strength, we repeated the Cox models with each of these terms alone and with both terms together. In further analyses we added a term for gait speed, an alternative measure of muscle function.

Since sarcopenia and physical frailty share common motor performances, we examined the inter-relationship of their individual components in a correlation matrix. Then we employed principal component analysis to determine if there was evidence that their components manifest distinct constructs based on their factor loading. We examined all six measures used to construct sarcopenia and physical frailty together in single Cox models to determine which components were independently associated with adverse health outcomes. Then we added interactions terms for age and sex. In further supporting analyses, we employed semiparametric proportional hazards models to examine potentially nonlinear effects of SMI and grip strength (**S1 Analysis and S2 Fig)**.

*A priori* level of statistical significance was 0.05. Models were examined graphically and analytically and assumptions were judged to be adequately met. Programming was done in SAS version 9.4 (SAS Institute Inc, Cary, NC) and in R-Studio (RStudio Inc, Boston, MA) [31, 32].

## Results

### Clinical characteristics of the analytic group

There were 1466 adults included in these analyses and their clinical characteristics at the study baseline are summarized in **Table 1**.

**Table 1:** Clinical characteristics of analytic cohort at baseline.

Measure	All	Female	Male
	Mean (SD) or N (%)	Mean (SD) or N (%)	Mean (SD) or N (%)
Number of persons	1466	1107 (75.5%)	359 (24.5%)
Baseline Age	81.3 (7.25)	81.1 (7.38)	82.0 (6.81)
Black Race	69 (4.7%)	56 (5.1%)	13 (3.6%)
Education (years)	15.3 (3.06)	14.9 (2.89)	16.4 (3.30)
Height (meters)	1.63 (0.10)	1.6 (0.07)	1.75 (0.08)
Muscle mass (kg)	16.9 (5.81)	14.1 (2.77)	25.4 (4.28)
Skeletal Muscle Index (SMI)	6.2 (1.59)	5.5 (0.96)	8.3 (1.29)
Grip strength (kg)	20.3 (8.32)	17.3 (5.70)	29.8 (8.04)
Gait speed(m/sec)	0.59 (0.20)	0.6 (0.19)	0.6 (0.20)
BMI (kg/m^2^)	27.3 (5.32)	27.2 (5.58)	27.5 (4.39)
Physical Activity (hrs/week)	3.4 (3.64)	3.3 (3.59)	3.9 (3.79)
Fatigue (0–2)			
none	1167 (79.6%)	861 (77.8%)	306 (85.5%)
1	212 (14.5%)	173 (15.6%)	39 (10.9%)
2	86 (5.9%)	73 (6.6%)	13 (3.6%)
Claudication	138 (9.4%)	109 (9.9%)	29 (8.1%)
Congestive heart failure	80 (5.5%)	62 (5.6%)	18 (5.0%)
Myocardial infarction	146 (10.0%)	84 (7.6%)	62 (17.3%)
Stroke	143 (9.8%)	107 (9.7%)	36 (10.0%)
Hypertension	830 (56.6%)	644 (58.2%)	186 (51.8%)
Smoking history	621 (42.4%)	439 (39.7%)	182 (50.7%)
Diabetes	198 (13.5%)	133 (12.0%)	65 (18.1%)
Cancer	551 (37.6%)	409 (37.0%)	142 (39.6%)
Total calories (kcal/day)	1755.9 (545.27)	1707.8 (519.98)	1898.9 (592.77)
Protein (% kcal/day)	16.9 (3.06)	17.1 (3.19)	16.3 (2.57)
Diet quality score (0–15)	7.9 (1.77)	8.0 (1.76)	7.7 (1.78)

### Continuous sarcopenia

Muscle mass and grip strength measures used to construct the continuous sarcopenia measure were weakly related (**Table 2).** The continuous sarcopenia measure was constructed so that the lower the score below 100% indicates more severe sarcopenia and scores ≥100 were individuals without sarcopenia. Overall, the mean continuous sarcopenia measure was 99.97% (SD = 21.03%), with 806 (55.0%) participants meeting criteria for sarcopenia at baseline.

**Table 2:** Correlations and principal component analysis (PCA) of measures used to construct sarcopenia and physical frailty metrics.

	Pearson Correlations					PCA	
Measure	GS	F	PA	BMI	MM	F1	F2
Gait Speed	0.36^**^**^	-0.19^**^**^	0.17^**^**^	0.13^**^**^	0.02	**0.73**	-0.03
Grip Strength (GS)	-	-0.18^**^**^	0.17^**^**^	0.13^**^**^	0.19^**^**^	**0.67**	0.33
Fatigue (F)		-	-0.16^**^**^	0.03	-0.07^**$**^	**0.57**	-0.18
Physical activity (PA)			-	-0.11^**^**^	0.04	**-0.55**	-0.01
Body Composition (BMI)				-	0.45^**^**^	-0.14	**0.82**
Muscle Mass (MM)					-	0.11	**0.78**

**$** <0.05

^<0.001; GS: grip strength; F: fatigue; PA: physical activity; BMI: body composition; MM: muscle mass; PCA: principal component analysis; F1: factor 1; F2: factor 2

Continuous sarcopenia was associated with age (r = 0.34, p<0.001and with education (r = 0.15, p < .001); men were less sarcopenic [men: 107.5 (SD = 20.6) than women: 97.54 (SD = 20.60); t_580_ = -21.98, p< 0.001. Continuous sarcopenia was modestly related to diet quality score (r = 0.10, p = 0.006) but not to total daily calories r = 0.01, p = 0.850) or daily percent calories from protein consumption r<0.00, p = 0.998).

**Fig 1** shows the heterogeneity of muscle mass and motor function used to construct continuous sarcopenia even within the binary sarcopenia classification employed in prior studies [14]. By definition, the distributions of both muscle mass and of grip strength are truncated in the sarcopenic group. Among non-sarcopenic participants, grip strength was generally above the cut-off, but SMI showed substantial overlap with the sarcopenic group. Gait speed, another measure of motor function, of the non-sarcopenic group also shows overlap with the sarcopenic group. Due to way the continuous sarcopenia was constructed, it was strongly correlated with the binary sarcopenia variable employed in prior studies (r = -0.789, p<0.001).

### Continuous sarcopenia and adverse health outcomes

Prior cross-sectional studies suggest that sarcopenia is associated with adverse health outcomes [22]. Cox proportional hazard models that included terms for age, sex, education, and race were employed to examine the associations of continuous sarcopenia with adverse health outcomes. A higher level of baseline continuous sarcopenia, reflecting less sarcopenia, was associated with reduced risk of death and decreased risk of subsequent ADL, IADL and mobility disability (**Table 3**).

**Table 3:** Continuous sarcopenia and incident adverse health outcomes.

Model Terms	Mortality	IADL	ADL Disability	Mobility Disability
Persons in model	1466	534	1026	592
Number of outcomes	579	343	483	363
Years of follow-up	5.6 (3.8)	3.7 (2.8)	4.8 (3.2)	3.9 (3.0)
Age	1.10 (1.09, 1.12) **^**	1.07 (1.05, 1.10) **^**	1.10 (1.08, 1.12) **^**	1.07 (1.05, 1.09) **^**
Male Sex	1.72 (1.42, 2.09) **^**	0.75 (0.56,1.01)	0.79 (0.61, 1.01)	0.85 (0.64, 1.13)
Education	0.97 (0.94, 1.00) ^$^	0.99 (0.95, 1.03)	0.99 (0.96, 1.02)	0.98 (0.94, 1.02)
Black race	1.59 (0.97, 2.62)	1.33 (0.75, 2.38)	0.94 (0.51, 1.74)	1.42 (0.78, 2.57)
Continuous Sarcopenia (%)	0.70 (0.62, 0.78) **^**	0.80 (0.70, 0.93) ^#^	0.81 (0.71, 0.91) **^**	0.81 (0.70, 0.93) ^#^
*Continuous Sarcopenia (%) (Excluded outcome <1yr)	0.70 (0.62, 0.79) **^** 30/579 (5.2%)	0.86 (0.73, 1.02) 115/343 (33.5%)	0.81 (0.70, 0.92) **^** 97/483 (20.0%)	0.78 (0.66, 0.92) ^#^ 116/363 (32.0%)

Each column represents a separate Cox model for a different adverse health outcome with model terms shown on the left. Participants were included for all analyses for which they had valid longitudinal data. Missing Data: IADL: (n = 113, 7.7%); ADL: n = 195, 13.3%); Mobility disability: (n = 122, 8.3%). To facilitate comparisons across different units, coefficients of continuous sarcopenia are per SD. The two rows below the primary models give c-statistics and 95% confidence intervals. All other cells are point estimates and confidence intervals for hazard ratios.

*The bottom row shows the results of a sensitivity analysis for the same models shown above but excluding cases which developed the outcome shown at the top of the column during the first year of follow-up. The number of cases excluded and its percentage of the total number of incident cases in the primary model is shown in the same cell. p-Value for terms in each cell-**$**<0.05

^**#**^<0.01

**^**<0.001

Chronic health conditions can affect both motor and muscle mass. These results were unchanged when controlling for eight chronic health conditions including: cancer, three vascular disease risk factors (hypertension, smoking and diabetes) and four vascular diseases (claudication, congestive heart disease, myocardial infarction and stroke).

Body composition and muscle mass can be affected by nutritional status [33]. The associations of sarcopenia and adverse health outcomes were also unchanged when we added terms to these models to control for nutritional status based on total daily calories intake, daily percent calories from protein consumption and diet quality score (**S2 Table**).

In sensitivity analyses, we repeated the models excluding individuals who developed incident outcomes within the first year of follow-up. The point estimates for the associations of sarcopenia with adverse health outcomes were unchanged (**Table 3**).

Models with interaction terms added to the previous models demonstrated that the associations of sarcopenia and adverse health outcomes did not vary with age or sex (results not shown).

### Components of continuous sarcopenia and adverse health outcomes

To leverage the advantages of a composite metric, its individual components should be related to one another. If its components are not adequately correlated with one another, using the constructed composite may obscure rather than highlight important associations of its components. Continuous sarcopenia was constructed from grip strength and lean muscle mass. Grip strength and lean muscle mass are only modestly correlated with a shared variance less than 4% (r^2^), and they load on different factors (**Table 2**).

Therefore, we repeated the previous Cox models including terms for each component alone and together to determine the strength of the associations with adverse health outcomes. **Model 1 (Table 4)** shows that grip strength alone is strongly related to all 4 outcomes (p-values all<0.001). Gait speed is also related to all four outcomes when controlling for grip strength (**Table 4, Model 4**).

**Table 4:** Associations of measures used to construct sarcopenia and physical frailty in relation to adverse health outcomes.

Model	Model Terms	Mortality	IADL Disability	ADL Disability	Mobility Disability
**1**	Grip Strength	0.68 (0.61, 0.76) ^	0.68 (0.57, 0.80) ^	0.66 (0.58, 0.75) ^	0.72 (0.62, 0.85) ^
**2**	Muscle Mass	0.87 (0.78, 0.96) ^#^	0.95 (0.82, 1.10)	1.12 (1.00, 1.24) ^$^	1.04 (0.90, 1.19)
**3**	Grip Strength	0.69 (0.62, 0.77) ^	0.68 (0.57, 0.80) ^	0.65 (0.57, 0.74) ^	0.72 (0.61, 0.84)
	Muscle Mass	0.91 (0.82, 1.01)	0.99 (0.85, 1.14)	1.16 (1.05, 1.29) ^$^	1.07 (0.93, 1.23)
**4**	Grip Strength	0.76 (0.68, 0.85) ^	0.69 (0.58, 0.82) ^	0.71 (0.62, 0.81) ^	0.73 (0.62, 0.86) ^
	Gait speed	0.71 (0.64, 0.78) ^	0.78 (0.68, 0.90) ^	0.65 (0.58, 0.74) ^	0.74 (0.64, 0.84) ^
**5**	Grip Strength	0.78 (0.69, 0.87) ^	0.69 (0.58, 0.82) ^	0.69 (0.61, 0.79) ^	0.73 (0.62, 0.85) ^
	Gait speed	0.69 (0.63, 0.77) ^	0.78 (0.68, 0.90) ^	0.66 (0.58, 0.75) ^	0.74 (0.65, 0.85) ^
	Muscle Mass	0.87 (0.78, 0.96) ^#^	0.97 (0.84, 1.12)	1.15 (1.03, 1.28) ^$^	1.05 (0.91, 1.20)
**6**	Grip Strength	0.80 (0.70, 0.90) ^	0.72 (0.61, 0.86) ^	0.72 (0.62, 0.82) ^	0.76 (0.65, 0.90) ^
	Gait speed	0.72 (0.65, 0.80) ^	0.80 (0.70, 0.92) ^#^	0.68 (0.60, 0.78) ^	0.75 (0.66, 0.86) ^
	Muscle Mass	0.87 (0.77, 0.98) ^#^	0.90 (0.75, 1.08)	1.05 (0.92, 1.19)	0.95 (0.80, 1.13)
	BMI	0.98 (0.88, 1.09)	1.11 (0.93, 1.34)	1.22 (1.07, 1.40) ^#^	1.24 (1.04, 1.47) ^$^
	Fatigue	1.14 (1.05, 1.23) ^#^	1.25 (1.04, 1.51) ^$^	1.13 (1.01, 1.20) ^$^	1.15 (0.97, 1.36)
	Physical Activity	0.92 (0.83, 1.03)	0.93 (0.82, 1.05)	0.95 (0.85, 1.06)	0.86 (0.76, 0.97) ^$^

Each cell in the top of the table shows the association [Hazards Ratio, (95% confidence interval)] of the measure (Model Terms) which were included in series of six Cox models (Model) which sequentially add different measures used to construct sarcopenia and physical frailty with the adverse health outcome shown at the top of each of column. **Models 1–3** shows the results for measures of sarcopenia, grip strength and muscle mass, alone and together. **Model 4** shows the results for two measures of motor function together grip strength and gait speed used by some investigators for characterizing motor function for sarcopenia. **Model 5** adds muscle mass to both measures of motor function used by some for constructing binary sarcopenia. **Model 6** shows the results for all six measures used by many investigators to construct sarcopenia and physical frailty. To facilitate comparisons across different units, coefficients are per SD of the relevant component measure. All models also included four additional terms for age, sex, education, and black race; these terms are not shown. In each cell the following symbols effect the statistical significance of each parameter **$**<0.05

**#**<0.01

**^** ≤0.001.

Lean muscle mass alone (**Table 4, Model 2**) or together with grip strength and gait speed (**Table 4, Model 3 and 5**) is only weakly associated with mortality and disability. These models (**Table 4, Models 1–5**) suggest that motor function rather than lean muscle mass drives the associations with all four adverse health outcomes.

### Components of sarcopenia and physical frailty and adverse health outcomes

Instruments assessing physical frailty and sarcopenia include measures which assess motor function (grip strength and gait speed) and muscle bulk (lean muscle mass or BMI). In further analyses, we examined the inter-relationship and the contributions of the individual measures used for their construction to test the hypothesis that lean muscle mass was independently associated with adverse health outcomes. Composite physical frailty was constructed so that higher scores correspond to more severe physical frailty. Composite frailty has a roughly symmetric distribution in our group, with mean = 0.01; SD = 0.55 [10% percentile = -0.66, 90% percentile = 0.79].

Continuous sarcopenia and composite physical frailty were strongly correlated (r = 0.66, p = <0.001), as illustrated in **S3 Fig**. **Table 2** shows the correlations among the six measures used to construct composite measures of sarcopenia and physical frailty. Principal component analysis of these 6 measures yielded two factors with eigenvalues of greater than 1 accounting for about 51% of the total variation. Following rotation of these factors, body composition measures (muscle mass and BMI) loaded on one factor and the other 4 clinical measures loaded on a second factor.

Grip strength and gait speed were independently associated with all four adverse health outcome when included in a single model (**Table 4, Model 4**). Lean muscle mass did not make an independent contribution to adverse health outcomes when controlling for motor function (**Table 4, Models 5 vs 4**). Next, we added terms for BMI, fatigue and physical activity which are used to construct physical frailty. In contrast to lean muscle mass, BMI, fatigue and physical activity, grip strength and gait speed were associated with all four adverse outcomes (**Table 4, Model 6**).

## Discussion

Identifying adults at risk for distinct and specific adverse health outcomes is a public health priority to facilitate early interventions. Much research over the past two decades has suggested that assessment of sarcopenia based on the combined assessment of muscle structure and function could identify older adults at risk for adverse health outcomes. Similarly, physical frailty, another commonly studied aging phenotype based in part on grip strength and gait speed, has been reported to identify adults at risk for adverse health outcomes.

This study describes a continuous measure of sarcopenia constructed from measures of grip strength and muscle mass derived from BIA. Continuous sarcopenia was strongly associated with survival, risk of incident IADL, ADL and mobility disability in about 1500 older adults. We then examined the individual measures used to construct sarcopenia and physical frailty. These analyses showed that lean muscle mass metrics based on bioimpedance used to categorize adults with sarcopenia, and BMI, fatigue and physical activity used to identify adults with physical frailty, were not associated with adverse health outcomes in older adults when grip strength and gait speed were included in the model. This suggests that impaired motor function is the primary driver of the associations attributed to sarcopenia and physical frailty with adverse health outcomes. It is important to note that grip strength and gait speed were robust, but non-specific drivers of all four adverse health outcomes examined in the current study. Together these findings emphasize the limitations of current muscle structure metrics i.e. lean muscle mass as well as muscle function based on grip strength and gait speed in predicting specific adverse health outcomes. Further work is needed to identify other facets of muscle structure as well as additional motor function metrics which together can be used to identify adults at risk for specific adverse health outcomes.

It has long been recognized that aging is associated with loss of muscle mass and diverse motor abilities. It was also recognized that despite the utility of BMI as a risk factor for adverse health outcomes, changes in BMI do not distinguish between changes in muscle mass and fat. Previous measures of muscle mass were limited to specialized lab setting for imaging or physiologic assessments of the percentage of body muscle mass versus fat. Employment of electrical bioimpendance has facilitated the collection of measures of muscle mass together with motor function on a much larger range of old adults. As these data have accumulated, there has been a growing recognition that motor function may be the primary determinant of sarcopenia’s association with adverse health outcomes [2, 4, 34–37].

The continuous measure of sarcopenia developed in this study was associated with adverse health outcomes and may circumvent some of the limitations of categorical measures to capture the heterogeneity of motor decline with better fidelity. However, our analyses of the components used to identify sarcopenia, showed that grip strength and gait speed alone or together, rather than lean muscle mass, are the primary drivers of the associations of sarcopenia with adverse health outcomes. These findings are consistent with prior studies which have suggested that motor function rather than muscle mass drives the association with mortality [38]. These data lend further support for the growing recognition that motor function and not lean muscle mass may be the primary determinant of sarcopenia’s association with adverse health outcomes [4].

The previous findings led us to examine the individual components of another common aging phenotype, physical frailty which, like sarcopenia, includes measures of grip strength and gait speed in its construction by many investigators [9]. Sarcopenia and physical frailty are usually examined separately and it is not common to compare the individual components used in their construction simultaneously in the same individuals as we have done in the current study. Our findings lend empiric support for the notion in the prior literature that sarcopenia and physical frailty are related phenotypes [39, 40]. Three aspects of these analyses raise questions as to what extent sarcopenia and physical frailty are distinct from each other and to what extent they make independent contributions for predicting adverse health outcomes when controlling for motor function in older adults. First, the individual components used to construct both phenotypes loaded on the same two factors, a motor function and body composition factor. Second, in a series of complementary analyses which sequentially added each of the components used to construct both phenotypes, motor function i.e., grip strength and gait speed rather than the other covariates, were the terms most consistently associated with adverse health outcomes. These findings that motor function is the primary driver of the associations of both sarcopenia and physical frailty with adverse health outcomes in older adults raises a question about the utility of these labels and emphasizes the need for empiric data to justify the construction of different aging phenotypes.

Grip strength and gait speed which capture different facets of motor function are robust, but non-specific predictors of adverse health outcomes [36, 41, 42]. Thus, even if one were to exclude lean muscle mass from the phenotype of sarcopenia, the combination of grip strength and gait speed together, as proxies of overall motor function, still lack the necessary specificity for risk stratification of older adults for distinct adverse health outcomes. So, for example it is still not possible to determine which adults will develop a specific disability or is at high risk for death.

Considering multiple clinical domains as a single complex phenotype may have important clinical and research implications, as geriatric interventions are increasingly multi-modal, targeting motor, cognitive and psychosocial components of aging [43–45]. Yet, our prior work has shown that due to its complexity, assessment of several motor performances or behaviors may be needed to adequately capture motor function and its heterogeneous decline in older adults [22, 46]. Moreover, the varied pathologies and degenerative changes that can affect the distributed motor pathways add additional complexity and contribute to the phenotypic heterogeneity of late-life motor decline [7, 47]. There is also increasing attention on the role of nutritional status and its contribution to motor function and muscle mass in older adults [48]. Yet, despite these difficulties, measuring different facets of motor performances is critical to facilitate public health efforts to elucidate distinctive motor risk profiles that identify adults at risk for specific adverse health outcomes. More granular measures of motor function which can be derived from unobtrusive sensors may not be sufficient for identifying individuals at risk for distinct adverse health outcomes [46]. Interrogating motor subsystems, like muscle structure, which are crucial for healthy motor behavior may yield measures which can be combined with motor function metrics to improve risk stratification for distinct adverse health outcomes.

This study has some limitations. Our study is restricted to very old adults whose average gait speed is very slow and may not inform on sarcopenia in the full spectrum of older adults. Participants in the current study were willing to provide organ donation at the time of their death, which could have introduced bias. The mostly white participants have more years of education than the general population, highlighting the need to replicate these findings in other cohorts. As with other studies of muscle mass derived from BIA, these measures may not provide the precision of other modalities for measuring muscle mass and we did not control for participant’s water level at the time of exam. However, several factors increase confidence in the findings from this study. These findings are based on a large number of well-characterized older persons at baseline. MAP achieves high rates of follow-up, increasing internal validity. The components used to construct sarcopenia and physical frailty were based on uniform structured procedures.

## Conclusions

The results from the current study suggest that impaired motor function is the primary driver of associations with adverse health outcomes, rather than lean muscle mass used to construct sarcopenia or other non-motor covariates used to construct physical frailty. These emphasize that importance of joint modeling of different covariates to determine the primary drivers of adverse health outcomes. Nonetheless, while, these results highlight the important contribution of impaired motor function to adverse health in old age, the current study also shows that grip and gait are robust non-specific predictors of the four adverse outcomes examined in this study. So, these motor performances, alone or together, are not sufficient to identify older adults at risk for specific outcomes i.e., a specific disability or increased risk for death. Consequently, the current study highlights that further work is needed fill our knowledge gaps about both muscle structure as well as motor function in order to identify older adults at risk for specific adverse health outcomes.

To fill these knowledge gaps, it is important to note that muscle structure provides two unique health perspectives via the distinct morphologic changes in muscle fiber types and size and fiber groupings manifested by systemic diseases i.e., metabolic disorders, poor nutritional status and chronic health conditions diseases versus CNS diseases. Moreover, many vital morphologic features of muscle are influenced by CNS structures distributed in structures extending from the brain to spinal motor neurons [1]. Few studies have systematically investigated muscle morphology and its underlying molecular mechanisms in large numbers of well-characterized older adults and none have linked morphologic changes in muscle with degeneration of nerve, spinal cord and brain in the same older individuals. While muscle and even nerve can be obtained in living older adults, the influences of both muscle and CNS degeneration can only be studied together in tissues obtained from well-characterized older individuals at the time of death. Additionally, the rapid advances in sensor technology offers investigators the opportunity to fill knowledge gaps about muscle function by extracting more granular motor metrics. Combining novel muscle structure indices together with more granular motor function metrics has potential to advance aging research and clinical care of older adults by providing risk models that can identify older adults at risk for specific adverse health outcomes.

## Supporting information

S1 Fig Sex-specific skeletal muscle index (SMI) and grip strength. (PDF)

S2 Fig Semiparametric models of grip strength and SMI. (PDF)

S3 Fig Association of continuous sarcopenia and composite physical frailty. (PDF)

S1 Table Composite sarcopenia average and incident adverse health outcomes. (PDF)

S2 Table Continuous sarcopenia average and incident adverse health outcomes (adjusted). (PDF)

S1 Method Calculation of sarcopenia measures. (PDF)

S1 Analysis Semiparametric models. (PDF)
