# Supplementary material for: Motor function is the primary driver of the associations of sarcopenia and physical frailty with adverse health outcomes in community-dwelling older adults
Source: PLoS One. 2021 Feb 2;16(2):e0245680. doi: 10.1371/journal.pone.0245680 (PMC7853482; doi:10.1371/journal.pone.0245680)
Supplement: S1 Fig — (PDF) [file pone.0245680.s001.pdf]

S1 Fig. Sex-specific skeletal muscle index (SMI) and grip strength

This figure which shows Skeletal Muscle Index (SMI, Y axis) versus Grip strength (X axis) separately for females and for males. The units on the right and top axes are percentages of sex-specific thresholds (grip strength; 20 kg (Female) and 30 kg(male) and SMI (6.42 (Female) and 8.87 (Male). For example, a female with grip strength 25 kg (125% of threshold) and SMI= 7.5 (117% of threshold) would have a continuous sarcopenia of 125%, and would not meet criteria for binary sarcopenia. A male with the same values (25 kg, or 83% of threshold and 7.5, 85% of threshold)) would have a continuous sarcopenia of 85%, and would meet the criteria for binary sarcopenia. The lower quadrant outlined in black includes those who have continuous Sarcopenia <100%. The participants whose values are inside the box (to the lower left in each panel) are the people deemed to have binary sarcopenia. A person whose values were both exactly at threshold would have percentages of exactly 100%, or continuous sarcopenia of 100%, which does not qualify as binary sarcopenia.

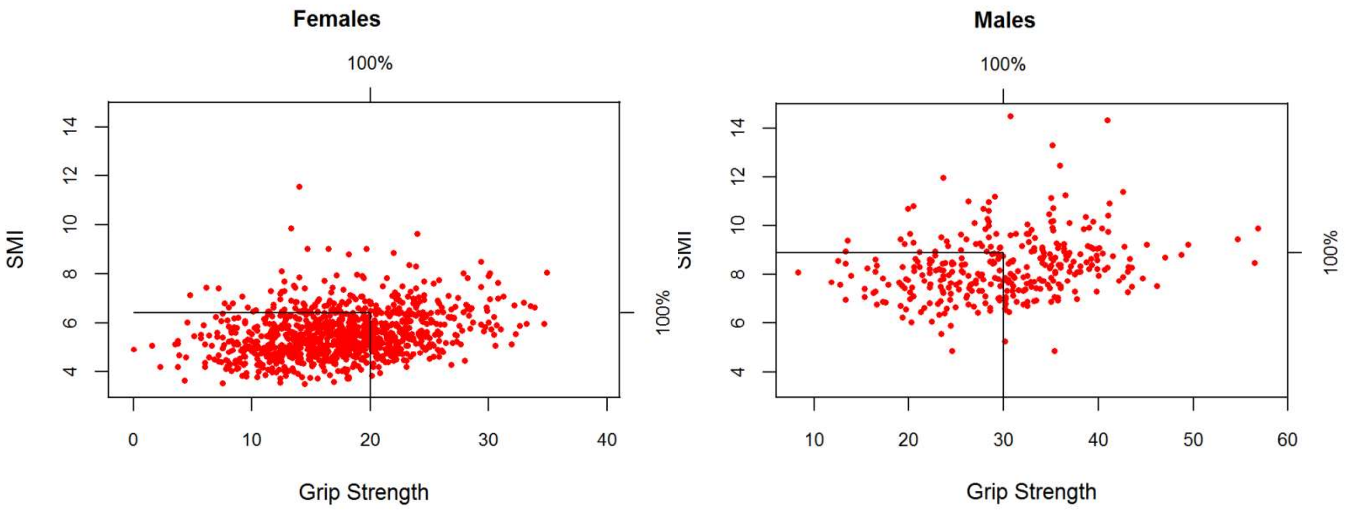

.
