# Supplementary material for: Motor function is the primary driver of the associations of sarcopenia and physical frailty with adverse health outcomes in community-dwelling older adults
Source: PLoS One. 2021 Feb 2;16(2):e0245680. doi: 10.1371/journal.pone.0245680 (PMC7853482; doi:10.1371/journal.pone.0245680)

S2 Fig. Semiparametric Models of grip strength and SMI.
The panels here show the estimated functions of SMI (A) and Grip strength (B) when the model shown above is fitted. The estimated functions are shown as connected circles (each circle represents a participant in the sample); confidence intervals are shown as dotted lines. These functions show that grip strength has a strong, largely linear, and well-supported association with mortality and that the association of SMI is nonlinear and weaker.

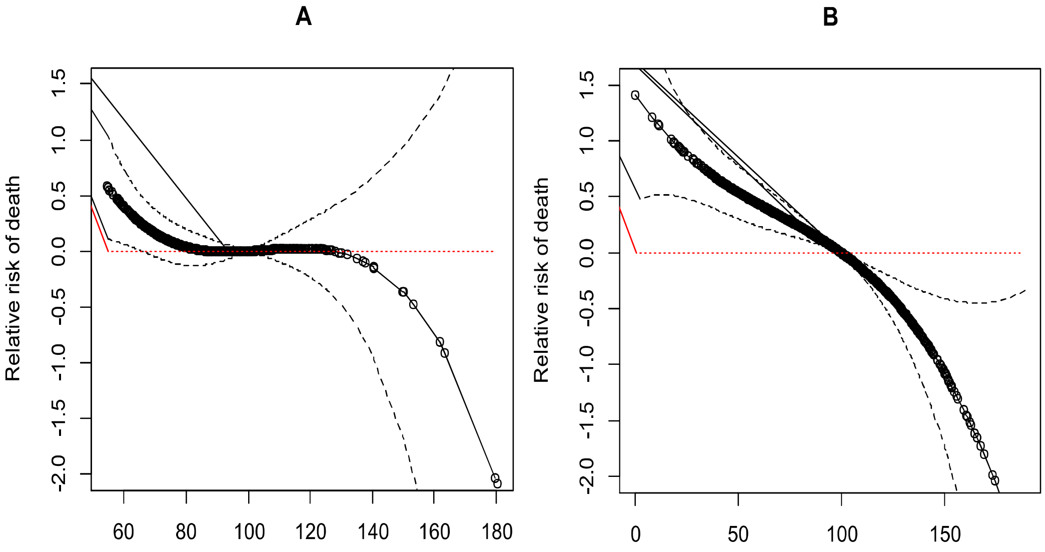

Supplement: S2 Fig — (PDF) [file pone.0245680.s002.pdf]
