# Supplementary figures and images for: Motor function is the primary driver of the associations of sarcopenia and physical frailty with adverse health outcomes in community-dwelling older adults
Source: PLoS One. 2021 Feb 2;16(2):e0245680. doi: 10.1371/journal.pone.0245680 (PMC7853482; doi:10.1371/journal.pone.0245680)

**S3 Fig. Association of continuous sarcopenia and composite physical frailty**

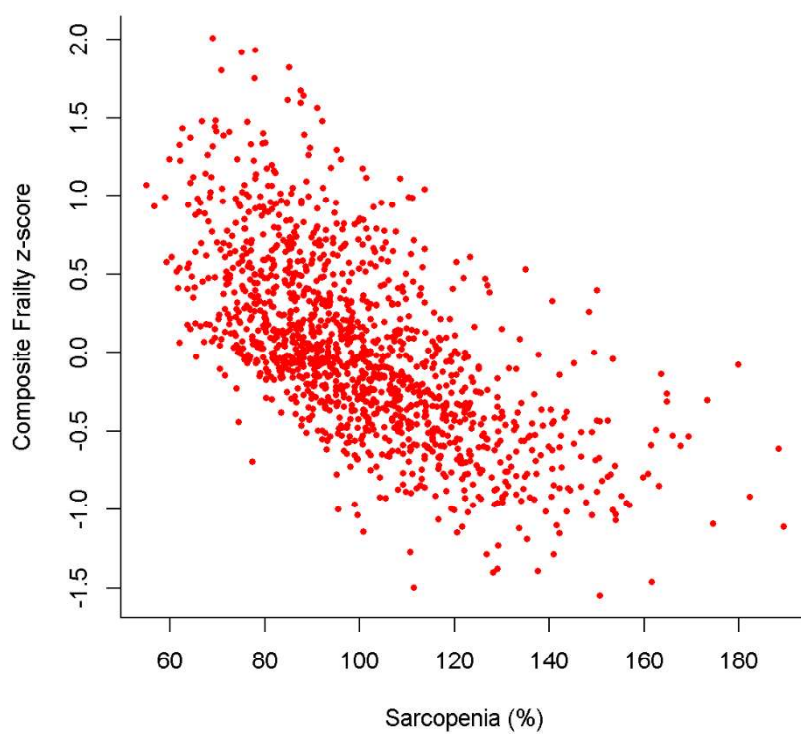

Supplement: S3 Fig — (PDF) [file pone.0245680.s003.pdf]
