# Supplementary material for: Motor function is the primary driver of the associations of sarcopenia and physical frailty with adverse health outcomes in community-dwelling older adults
Source: PLoS One. 2021 Feb 2;16(2):e0245680. doi: 10.1371/journal.pone.0245680 (PMC7853482; doi:10.1371/journal.pone.0245680)
Supplement: S1 Table — (PDF) [file pone.0245680.s004.pdf]

S1 Table. Composite Sarcopenia Average and Incident Adverse Health Outcomes.

This table is similar to Table 3 in the main text, except that it shows the associations of composite sarcopenia average with adverse health outcomes while Table 3 in the main text shows the results for the continuous sarcopenia measure. Findings for composite sarcopenia average are very similar to those for continuous sarcopenia, except that the effects of male sex are more pronounced for this measure. & <0.05, #<0.01; ^<0.001

Model Terms	Mortality	IADL	ADL Disability	Mobility Disability
Persons in model	1465	534	1025	592
Number of outcomes	579	343	482	363
Years of follow-up	5.6 (3.8)	3.7 (2.8)	4.8 (3.2)	3.9 (3.0)
Age	1.1.11<br>(1.09, 1.13)[^]	1.08<br>(1.05, 1.10)[^]	1.11<br>(1.09, 1.13)[^]	1.07<br>(1.05, 1.09)[^]
Male Sex	0.89<br>(0.70, 1.12)	0.48<br>(0.34,0.67)[^]	0.58<br>(0.43, 0.76)[^]	0.60<br>(0.44, 0.83)[#]
Education	0.97<br>(0.94, 1.00)[&]	0.99<br>(0.94, 1.03)	0.99<br>(0.95, 1.02)	0.98<br>(0.94, 1.02)
Black race	1.58<br>(0.96, 2.62)	1.36<br>(0.76, 2.43)	0.91<br>(0.50, 1.68)	1.44<br>(0.79, 2.61)
Composite Sarcopenia (%)	0.65<br>(0.57, 0.74)^	0.73<br>(0.60, 0.88)[#]	0.84<br>(0.73, 0.98)^	0.82<br>(0.68, 0.90)[&]
