# Supplementary material for: Motor function is the primary driver of the associations of sarcopenia and physical frailty with adverse health outcomes in community-dwelling older adults
Source: PLoS One. 2021 Feb 2;16(2):e0245680. doi: 10.1371/journal.pone.0245680 (PMC7853482; doi:10.1371/journal.pone.0245680)
Supplement: S2 Table — (PDF) [file pone.0245680.s005.pdf]

S2 Table. Continuous Sarcopenia Average and Incident Adverse Health Outcomes (adjusted). This table cell shows the term of the association of composite sarcopenia with each of the four adverse health outcomes obtained from a Cox model which included terms for age, sex, education and race (not shown). The upper row shows the results from the models unadjusted for chronic health conditions or diet measures. The next row shows that same model which included 3 additional terms to adjust for a history of cancer, and the sum of the three vascular risk factors (hypertensions, smoking and diabetes) and four vascular diseases (claudication, congestive heart failure, myocardial infarction and stroke) Each of the next three rows added a term for a different diet measure to the unadjusted model shown in the top row. & <0.05, #<0.01; ^<0.001**.**

**Model Terms**	**Mortality**	**IADL**	**ADL Disability**	**Mobility Disability**
Unadjusted for health or diet	0.70 (0.62, 0.78)^	0.80 (0.70, 0.93)[#]	0.81 (0.71, 0.91)^	0.81 (0.70, 0.93)[#]
Adjusted for health conditions	0.70 (0.62, 0.78)^	0.80 (0.70, 0.92)[#]	0.81 (0.71, 0.91)^	0.81 (0.71, 0.93)[#]
Adjusted for diet score	0.71 (0.62, 0.82)^	0.79 (0.65, 0.95)[#]	0.79 (0.68, 0.92)[#]	0.79 (0.67, 0.95)[&]
Adjusted for protein	0.69 (0.61,0.79)^	0.77 (0.66,0.91)[#]	0.79 (0.69,0.90)^	0.77 (0.66,0.90)[#]
Adjusted for calories	0.68 (0.60, 0.78)^	0.77 (0.65, 0.91)[#]	0.78 (0.68, 0.90)^	0.77 (0.66, 0.90)^
