# Supplementary material for: Motor function is the primary driver of the associations of sarcopenia and physical frailty with adverse health outcomes in community-dwelling older adults
Source: PLoS One. 2021 Feb 2;16(2):e0245680. doi: 10.1371/journal.pone.0245680 (PMC7853482; doi:10.1371/journal.pone.0245680)
Supplement: S1 Method — (PDF) [file pone.0245680.s006.pdf]

S1 Methods. Calculation of Sarcopenia Measures

***Continous Sarcopenia*:** This measure is calculated as the maximum of skeletal muscle index and grip strength, each expressed as a percentage of the corresponding sex-specific threshold. The calculation is illustrated in **S1 Figure**. Composite Sarcopenia Average: composite sarcopenia average is calculated as the mean sex-specific z-score, where z-scores for SMI and for Grip strength are computed separately for men and for women, with means and SDs as shown in Table 1. For the example above (grip strength 25 kg and SMI=7.5), the female would have a composite sarcopenia average of 1.72 and the male would have a score of -0.61. That is, a female with these values would be less sarcopenic than a male. With difference reference values (grip strength=21 and SMI=4.5), the continuous sarcopenia would be 105% for a female participant and the composite sarcopenia average would be -0.20, because the below average SMI (z-score contribution -1.04) would dominate the above average grip strength (z-score contribution 0.65). The sex-specific correlations of grip strength and SMI are not particularly high, so averaging these quantities does not improve the estimation of an underlying construct.

Composite Sarcopenia Average: The composite sarcopenia average is calculated as the mean sex-specific z-score, where z-scores for SMI and for Grip strength are computed separately for men and for women, with means and SDs as shown in Table 1. For the example above (grip strength 25 kg and SMI=7.5), the female would have a composite sarcopenia average of 1.72 and the male would have a score of -0.61. That is, a female with these values would be less sarcopenic than a male. With difference reference values (grip strength=21 and SMI=4.5), the continuous sarcopenia would be 105% for a female participant and the composite sarcopenia average would be -0.20, because the below average SMI (z-score contribution -1.04) would dominate the above average grip strength (z-score contribution 0.65). The sex-specific correlations of grip strength and SMI are not particularly high, so that averaging these two quantities is not improving the estimation of an underlying construct.
