# Supplementary material for: Motor function is the primary driver of the associations of sarcopenia and physical frailty with adverse health outcomes in community-dwelling older adults
Source: PLoS One. 2021 Feb 2;16(2):e0245680. doi: 10.1371/journal.pone.0245680 (PMC7853482; doi:10.1371/journal.pone.0245680)
Supplement: S1 Analysis — (PDF) [file pone.0245680.s007.pdf]

S1. Supporting analyses. Semiparametric models.

These analyses were undertaken to expand upon our findings that grip strength has stronger associations with incident adverse health outcomes than skeletal muscle index (SMI) does. Our goal is to check whether the two measures had nonlinear assocations with adverse health outcomes, because nonlinear associations could compromise our findings. We utilized R package survival (R Core Team, 2019) to fit Cox models for mortality, incident IADL disability, incident ADL disability, and incident mobility disability in which the effects of SMI and GRIP strength are modeled as nonlinear functions:

$$\lambda_i(t) = \lambda_0(t) \times \exp(\beta_1 \times age_i + \beta_2 \times msex_i + \beta_3 \times educ_i + f_1(SMI) + f_2(GRIP)),$$

where $\lambda_0(t)$ is the baseline hazard function, $f_1(SMI)$ and $f_2(GRIP)$ are 2 different nonlinear functions approximated by spline expansions (Gray, 1992 as implemented in Therneau, 2019). Negative values for the nonlinear functions correspond to protective associations with the outcome; positive values for the nonlinear functions correspond to higher hazards for the outcome. **S2 Figure** shows the functions estimated for mortality. These functions show that grip strength has a strong, largely linear, and well-supported association with mortality and that the association of SMI is nonlinear and weaker.

We explain these summaries in more detail:

Grip Strength. The function for grip strength (as percentage of the sex-specific threshold) resembles a line, though the function bends down for very large grip strengths. That is, low grip strength is associated with higher mortality and high grip strength is protective. The confidence bands are narrow, demonstrating that the data strongly support the estimated function. Particularly high grip strength is slightly more protective than a linear fit would suggest.

Skeletal Muscle Index: . For low values of SMI (< 70% or so), the function and its lower confidence limit are both positive, indicating an elevated risk of death for very low values of Skeletal Muscle Index.

The estimated function associated with SMI is flat and close to 0 for values of the composite sarcopenia between around 80% and 130%, so that SMI in this range is not associated with the hazard for mortality. For SMI > 150%, the function is negative, which would suggest that very high values of SMI are protective. However, the estimates of these spline functions can be unstable for near the edges of the data set, due to lack of observations (sparse circles), and the dotted lines representing confidence intervals are relatively far apart and surround 0 for SMI > 100%. Thus we conclude that the association of SMI with mortality is only evident for SMI substantially below the published cut-off value, and that even this association is weak. Together these semiparametric models of grip strength and SMI give results that are consistent with the parametric models shown in Table 3 in the text. Similar findings (not shown) were obtained for the other adverse health outcomes including IADL disability, ADL disability and mobility disability.

References

R Core Team 2019. R: A language and environment for statistical computing. R Foundation for Statistical Computing, Vienna, Austria. https://www.R-project.org.

Therneau, T. Spline terms in a Cox model. 2019. Self-published at: https://cran.r-project.org/web/packages/survival/vignettes/splines.pdf

Gray, RJ. Flexible Methods for Analyzing Survival Data Using Splines, With Applications to Breast Cancer Prognosis. Journal of the American Statistical Association 1992; 87: 942-951.
